# Validation of ageism scale for dental students in India: (Ageism Scale for Dental Students- India) – a cross sectional study.

**DOI:** 10.12688/f1000research.130685.1

**Published:** 2023-04-18

**Authors:** Ramya Shenoy, Praveen S Jodalli, Shushma Rao B, Aishwarya Shodhan Shetty, Manuel Thomas, Kamal Shigli, Leonardo Marchini

**Affiliations:** 1 Public of Publich Health Dentistry, Manipal College of Dental Sciences, Mangalore, Manipal Academy of Higher Education, Manipal, Karnataka, 575001, India; 2 Department of Conservative Dentistry, Manipal College of Dental Sciences, Mangalore, Manipal Academy of Higher Education, Manipal, Karnataka, 575001, India; 3 Department of Prosthodontics, DY Patil Dental School, Lohegaon, Pune, Maharashtra, 412105, India; 4 Comprehensive Care, School of Dental Medicine, Case Western Reserve University, Cleveland, Ohio, 44106, USA

**Keywords:** agesim, dental students, geriatric dentistry, oral health, validation

## Abstract

**Background**:

Ageism persists in many different societies as it is innate and subconscious in nature. Negative effects such as loneliness, mistreatment, and occupational discrimination are frequently present due to ageism. The dental students in our study were wary of the possible benefits of expensive dental care because ageism is rife in their field. There is no validated and reliable ageism scale to assess how dental students perceive ageism in India. The current study was carried out to validate the ageism scale for dental students in the Indian context.

Methods:

This was a cross sectional analytical study carried out among both males and females in Manipal College of Dental Sciences, Mangalore in which the instrument was 27-item Ageism scale for dental students. Content validity was done by six subject experts. The final version was administered to 213 students/Residents of dental school. The factorability of data was confirmed with KMO = 0.61 and Bartlett’s Test of Sphericity resulting in *p* < 0.001.

Results:

Final PCA model resulted in 15 items and six components that together accounted for 70.37% of overall variance. The six components had reliability ranging from marginal 0.51 (Component 6) to a high of 0.81 (Component 3). As per the gender differences by component females showed less ageism than men in “non-compliance” (-0.9(-1.66-0.14), p<0.05) and “practitioner perspective” (1.43 (0.84, 2.03), p<0.01). Statistical significance was seen in Barriers/concerns in dental treatment of elderly where residents showed reversed (1.4 (0.41, 2.38), p<0.01). Urban group showed more ageism for component ‘time restraint’ (-0.79 (-1.57, -0.02), p<0.05.

Conclusion:

Preliminary validation resulted in 15 item scale with six components with acceptable validity of the ageism scale and could be further tested in large samples. This scale will help recognize ageism in Indian context and provide necessary information to make changes in the curriculum as required.

## Introduction

Butler coined the phrase “Age-Ism” in 1961 to describe age discrimination, or prejudice between different age groups. ^1^ World Health Organization defines ageism as “the stereotyping, prejudice and discrimination toward people on the basis of age”. ^2^ Ageism is found to be a persistent factor across various cultures primarily because it is inherent and subconscious in nature. The negative effect of ageism is found to be more pronounced in older individuals. The common detrimental consequences of institutional ageism include discrimination in the workplace, old job applicants receiving fewer positive ratings than the young applicants, mistreatment, loneliness and patronising speech. ^3^^,^^4^ This can also be viewed as a consequence of negative portrayal of older people in media. ^5^^,^^6^

Aging, now being a global phenomenon due to the prevalence of chronic disease and multimorbidity among elderly patients, requires essential attention and care. This reveals the importance of understanding “ageism” which is pervasive among health care providers and institutions. It is found that “elderspeak” is often employed while communicating to older patients which can be related to negative effects for the geriatric patients as it results in lowering their self-esteem and confidence. ^7^ It also affects the treatment preferred, offered and provided. ^8^ Ageist attitudes fundamentally alter how problems are portrayed and the kinds of solutions that are put forth, which limits the development of acceptable policies about ageing. ^5^

There is also evidence that ageism is pervasive among the dental students. ^3^ Research by Veenstra *et al*. and Rucker *et al.* reveals the feeling of empathy and respect towards older patients was observed among dental students. ^9^^,^^10^ However, these students were apprehensive about the potential benefit of expensive dental treatment. Additionally, recent studies illustrate the students facing “patient compliance issues” and “preconceived notions about dental treatment” provided to older patients. ^11^ Moreover, dental colleges where separate gerodontology departments are not required as *per* regulatory body and the absence of non-emphasis of geriatrics in dental schools can lead to negative consequences. ^6^ To overcome such consequences, it is required to have culturally adaptive ageism scale for dental students to measure their perception. A valid and trustworthy ageism scale is not yet available to assess how dental students perceive ageism in India. In light of this, the current study was conducted to validate the ageism scale for dental students created in the United States (US) ^3^ to an Indian context by subject experts, final year dental undergraduate students and residents. It also intended to gauge if there are any differences among the male and female participants as observed in literature. ^3^^,^^10^ Differences in ageism in relation to other demographic data such as age, area of residence, year of study and history of living with elderly was also intended to be examined. The research objectives were to measure the construct validity, structural validity, and internal consistency of the ageism scale for dental students by final year undergraduate dental students and residents, as well as to quantify the content validity of the scale by subject experts.

## Methods

### Study design

This was a cross-sectional analytical study carried out in Manipal College of Dental Sciences, Mangalore. The mode of conducting was through online platform conducted concurrently from March 2021 to June 2021.

### Study population

Residents and final-year undergraduate dentistry students were the eligible participants for this study. The sample size for the present study was based on at least five individuals responding for each item while subjecting to factor analysis. ^12^ By implementing this method, the sample size would be 205. Following the universal sampling approach, the ageism scale for dental students was distributed to 250 final-year undergraduate dental students and residents after approval from the institution's head had been obtained in accordance with the inclusion criteria. The use of the universal sampling method eliminated the possibility of bias in the selection of participants. Finally, 213 students responded to the ageism scale. The method of self-reporting allowed for the identification of demographic information such as gender about the participants.

### Ethical considerations

Permission of Head of the Institution was taken. Institutional Ethics Committee clearance was obtained before commencement of the study (Protocol no. 21002, 9 ^th^ April 2021). Informed consent was electronically collected along with the responses to the ageism scale questionnaire, ^13^ and participation was completely voluntary. Confidentiality of the participants was maintained.

### Study instrument

Dental students' ageism scale: The initial version of the survey had a total of 27 items to measure ageism attitudes among dental students. A team of professors with experience in teaching geriatric dentistry from the United States and Europe created the original version. ^3^ A 27-item ageism scale specifically tailored for assessing ageism among dental students, which considers the specificities related to geriatric dentistry. The original version was tailored to developed countries health set –up. This is measured on six-point Likert scale (“Strongly disagree” = 1; “Disagree” = 2; “Slightly disagree” = 3; “Slightly agree” = 4; “Agree” = 5; “Strongly agree” = 6). The total score value is directly proportion to the understanding of the individual about ageism.

### Content validity by the subject experts

We asked six subject specialists with at least ten years of teaching experience and knowledge in working with senior citizens to take part in the study. None of these specialists were a part of the research team of this study. Through the online platform, the content was validated. The form's filling instructions was explained in detail through the information sheet. ^14^ The specialists were asked to review each item critically and were asked to provide score for each item from 1 = “The item is not relevant to the measured domain”; 2 = “The item is somewhat relevant to the measured domain”; 3 = “The item is quite relevant to the measured domain”; 4 = “The item is highly relevant to the measured domain” and written comment to improve the relevance of items. Once they had fully provided the scores on all the items, the experts were requested to submit their comments to the researcher. We all agreed that “Old age home/Senior citizen house/Senior dwelling” should be used in place of “nursing home” for Question 8 and Question 24. Since none of the other items on the agism scale made reference to where the elderly resided, none of the phrases in those 25 remaining questions needed to be modified. This was then administered to the 213 final-year undergraduate dentistry students and residents who were open to taking part in an online platform. They received detailed directions ^14^ on how to complete the electronic form.

### Data handling and statistical analysis

Statistical Package for Social Sciences (SPSS) version 26.0 (SPSS, Inc., Chicago, IL, USA) was used to calculate the results based on the data obtained. ^15^ Values obtained from the participants were computed to obtain descriptive and inferential statistics. A test of discriminant validity was conducted to look for differences based on gender, life history with senior people, year of study, and living in an urban or rural region. Mean and standard deviation was calculated for “age” and frequency with percentage was calculated for other demographic data. The Independent t test was used wherever applicable for the quantitative data obtained and participant responses which had missing data were excluded from the analysis. To measure item-scale correlation and inter item correlation Pearson correlation coefficient was used.

The reliability of the scale was evaluated using item analysis, internal consistency, and split-half test.

The goal of principal component analysis is to increase the sum of the squared loadings for each factor that is separately extracted. The loadings obtained from any other method of factoring would not explain as much variance as the principal component factor so, Principal component method of factor extraction used to carry out factor analysis for construct validity, structural validity, and internal consistency. In order to achieve what is referred to as “simple structure” in data, the varimax rotation approach is used. This method maximises the variance of the loadings inside each factor. Large loadings on a small number of variables describe the factors produced by the varimax rotations’ solution and increases the accuracy of construct validity, structural validity, and internal consistency while using the principal component method. ^16^ The Kaiser-Meyer-Olkin (KMO) test and Bartlett’s sphericity test was used to check the adequacy of the factorial analysis. A KMO greater than 0.60 and Bartlett’s significance *p* < 0.05 were considered acceptable.

Eigen values, also known as latent root in Principal Component method is the sum of squared values of factor loadings relating to a factor/component. ^16^ All components with Eigen values > 1.0 were initially extracted, and the number to keep was chosen using a combination of Scree Plot analysis and the overall variance that each component contributed. Items that (a) loaded poorly on any component or (b) cross-loaded across several components were removed as the Principal Component Analysis (PCA) was run iteratively. PCA was continued until the strongest and most parsimonious final set of items was reached. Cronbach's Alpha was used to assess the internal consistency of the final components, with a minimum acceptable value of 0.60 and an ideal value of 0.80. To identify items that were either possibly redundant due to multicollinearity (r|0.80|) or not meaningfully associated to any other item (max r|0.30|), correlation matrix of all 27 items was evaluated. *P* < 0.05 was considered to be the level of significance. The content validity of the scale was analysed using Content Validity Index (CVI). ^17^

*Calculating CVI* ^17^

There are two types of CVI: scale-based CVI and item-based CVI (S-CVI). There are two ways to calculate S-CVI: the proportion of items on the scale that receive a relevance value of 3 or 4 from all experts (S-CVI/UA), and the average of the I-CVI scores for all items on the scale (S-CVI/Ave). ^6^ The following table summarises the formula and definition of the CVI indices.

*The definition and formula of I-CVI, S-CVI/Ave and S-CVI/UA*.

**Table:** 

The CVI indices	Definition	Formula
**I-CVI** (item-level content validity index)	The proportion of content experts giving item a relevance rating of 3 or 4	**I-CVI** = (agreed item)/ (number of expert)
**S-CVI/Ave** (scale-level content validity index based on the average method)	The average of the I-CVI scores for all items on the scale or the average of proportion relevance judged by all experts. The proportion relevant is the average of relevance rating by individual expert.	**S-CVI/Ave** = (sum of I-CVI scores)/ (number of item) **S-CVI/Ave** = (sum of proportion relevance rating)/(Number of expert)
**S-CVI/UA** (scale-level content validity index based on the universal agreement method)	The proportion of items on the scale that achieve a relevance scale of 3 or 4 by all experts. Universal agreement (UA) score is given as 1 when the item achieved 100% experts in agreement, otherwise the UA score is given as 0.	**S-CVI/UA** = (sum of UA scores)/ (number of item)

The above-mentioned calculation led to the conclusion that I-CVI, S-CVI/Ave, and S-CVI/UA meet satisfactory levels, indicating that the scale of questionnaire’s content validity has been attained to a satisfactory level. When writing the report, this study used the STROBE checklist.

## Results

Of the 250 students a total of 213 undergraduate, including 30 male and 183 female, consented to take part in the study. Those participants who did not provide consent were not included in the study. Since this was a cross-sectional study, participation was limited to a single time point where questionnaires were sent electronically to all final year students and residents. Their age, gender and year of study were recorded along with history of living with elderly and urbanicity. The ‘semi urban’ was combined with ‘not urban’ as there was low sample size for that group ( Table 1).

**Table 1.** Demographic characteristics of study sample (N = 213).

Variable, Group	Frequency	Percent
Age (Mean, Standard Deviation)	22.26	1.29
Gender		
Male	30	14.1%
Female	183	85.9%
Year		
Intern	103	48.4%
Fourth-year-BDS	110	51.6%
History of living with elderly		
No	40	18.8%
Yes	173	81.2%
Urbanicity ^1^		
Not urban	43	20.2%
Urban	170	79.8%

^1^ Semi-urban was combined with “Not urban” due to low sample size.

### PCA results

First, all items were coded so that a higher value indicated *more* Ageism, so ten items were reverse-coded where 1 = “Strongly Agree” and 6 = “Strongly Disagree”. We indicate in our results whenever one of these items are mentioned that it was reverse coded for clarity.

There were no items that posed a risk of biasing the PCA results due to a multicollinear or insignificant relationship with other items. The factorability of the data was confirmed with KMO = 0.61 and Bartlett’s Test of Sphericity resulting in *p* < 0.001. The final PCA model resulted in 15 items and six components that together accounted for 70.37% of overall variance ( Table 2). The six components had reliability ranging from marginal 0.51 (Component 6) to a high of 0.81 (Component 3). While not ideal, the small number of items on each scale, which directly impacts Cronbach’s Alpha, may be the cause of these coefficients. ^18^

**Table 2.** Principal Components Analysis (PCA) for Indian Ageism Scale (15 items, α = 0.46).

Component (Cronbach’s Alpha), Item	Factor loading
Component 1 (α = 0.74) ***Barriers/Concerns in dental treatment of elderly***	
Q16 The elderly patient does not live long enough to make it worthwhile to invest time and effort in complex dental treatment	0.90
Q17 The elderly patient does not live long enough to make it worthwhile to invest money in expensive dental treatment	0.89
Q8 Elderly patients are better off in nursing homes	0.60
Q19 It is too costly to provide out of office dental care to homebound elderly patients	0.48
Component 2 (α = 0.68) ***Patient Compliance***	
Q5_R I tend to pay more attention towards my elderly patients than my younger patients	0.78
Q6_R I tend to have more sympathy towards my elderly patients than my younger patients	0.78
Q9_R Elderly patients tend to be more appreciative of the dental care I provide than younger patients	0.72
Component 3 (α = 0.81) ***Opinions of elderly***	
Q13 Elderly people do not take good care of their teeth	0.88
Q14 Elderly patients do not usually comply with dental advice	0.86
Component 4 (α = 0.70) ***Time consuming***	
Q3 Taking a medical History from elderly patients is frequently complex	0.87
Q2 Taking a medical History from elderly patients is frequently time-consuming	0.85
Component 5 (α = 0.67) ***Treatment success rate***	
Q21 Dental treatment is usually successful in elderly patients	0.89
Q12 My dental treatment planning for elderly patients is consistent with my middle-aged patients	0.75
Component 6 (α = 0.51) ***Practitioner perspective***	
Q24_R I would provide more homecare or nursing home dental treatment if I had more training	0.83
Q25_R Elderly patients should be treated by a someone with advanced training in geriatric dentistry	0.78

Statistically significant findings are highlighted in the tables for easy identification. Gender differences by component shows a statistically significant difference in scores on Component 4 where males (M = 6.93, SD = 2.08) scored 0.91 points lower than females (M = 7.84, SD = 1.94), p = 0.02 and Component 6 where Males (M = 5.93, SD = 1.66) scored 1.43 points higher on average, (95% CI = 0.84 – 2.03) than females (M = 4.5, SD = 1.5), *p* < 0.001 ( Table 3). Overall, there was one missing data when testing the gender differences and also 3 missing data for component 1, 2 and 5.

**Table 3.** Independent t-test for gender differences by component.

Component	Group mean (SD)		Mean difference (95% CI)	*p*-Value
	Male ( *n* = 30)	Female ( *n* = 182)		
Component 1	10.73 (3.63)	10.84 (3.64)	-0.11 (-1.53, 1.3)	0.877
Component 2	11 (2.65)	10.08 (3.18)	0.92 (-0.29, 2.13)	0.136
Component 3	7.17 (1.76)	7.43 (1.79)	-0.27 (-0.96, 0.43)	0.448
Component 4	6.93 (2.08)	7.84 (1.94)	-0.9 (-1.66, -0.14)	0.020
Component 5	7.1 (2.11)	6.78 (1.74)	0.32 (-0.38, 1.01)	0.372
Component 6	5.93 (1.66)	4.5 (1.5)	1.43 (0.84, 2.03)	0.000

*Note.* Female *n* = 180 for Components 1, 2, and 5.

Results of independent t- test for history of living with elderly based on the components had no statistically significant results ( Table 4). There was one missing data in this comparison and 3 missing data for component 1, 2 and 5. While comparing differences by component for year of study statistically there was significant difference in component one which was barriers/concerns in dental treatment of elderly. The residents (M = 11.56, SD = 3.89) and final year students (M = 10.16, SD = 3.26) had a mean difference of 1.4 (95% CI = 0.41 – 2.38) indicating residents had more barriers/concerns in treating the elderly (p = 0.06) and there were 3 missing data for component 1, 2 and 5.

**Table 4.** Independent t-tests for history of living with elderly differences by component.

Component	Group mean (SD)		Mean difference (95% CI)	*p*-Value
	No ( *n* = 40)	Yes ( *n* = 172)		
Component 1	11.49 (4.19)	10.68 (3.49)	0.81 (-0.46, 2.08)	0.211
Component 2	10.85 (3.39)	10.06 (3.04)	0.79 (-0.29, 1.86)	0.152
Component 3	7.3 (1.73)	7.42 (1.8)	-0.12 (-0.74, 0.5)	0.706
Component 4	7.85 (1.66)	7.67 (2.05)	0.18 (-0.51, 0.86)	0.614
Component 5	7.08 (1.76)	6.77 (1.8)	0.3 (-0.32, 0.93)	0.335
Component 6	5.05 (1.68)	4.62 (1.58)	0.43 (-0.12, 0.98)	0.128

*Note.* “No” *n* = 39 for Component 1, and “Yes” *n* = 171 for Component 1 and *n* = 170 for Components 2 and 5.

The students residing in urban areas (M = 7.86, SD = 1.86) felt that it was more time consuming to treat the elderly than when compared to those residing in not urban areas (M = 7.07, SD = 2.32) where the mean difference was 0.79 (p= 0.04) (95% CI = -1.57 - -0.02). Components 3, 4, and 6 each had one missing data, while 1, 2, and 5 each had three. All of the missing data entries were excluded for component-based analysis. Regardless of statistical significance, it is very important to note that the actual differences between groups are quite small, so results must be interpreted with caution.

## Discussion

The ageism dental scale, which was validated for the Indian context, produced a 15-item questionnaire distributed into the following six components; Barriers/Concerns in dental treatment of elderly, Patient Compliance, Opinions of elderly, Non-Compliance, Treatment success rate and Practitioner perspective. Due to the low loading of these questions over the components in principal component analysis, the remaining twelve questions—including questions 1, 4, 7, 10, 11, 15, 18, 20, 22, 23, 26 and 27—were omitted in accordance with the Indian context.

As per the gender differences by component females showed less agism as compared to men in the component of “Time consuming” and there was also statistically significant difference regarding “practitioner perspective”. A similar result was seen in validation study carried out in USA ^10^ whereas in the Greek version men showed more sympathy towards the elderly than women. ^19^ There was no difference in any of the components while comparing gender in the German ^20^ and Brazilian ^21^ versions whereas in the French version men scored significantly lesser than women in component three which dealt with education around older patients care. ^22^ Additionally, gender distinctions in factors 1 and 4 were discovered. Since they displayed significantly fewer ageist attitudes than male students in factor one (Q16 and Q17), female students tended to have greater compassion and empathy for the elderly; however, they found it significantly more difficult to obtain medical histories from elderly patients (Factor 4, Q2 and Q3). ^23^

There was no significant difference when it came to comparison between having history of living with elderly or not regarding all components. Similar results were seen with the USA, ^10^ German, ^20^ Brazil ^21^ and French versions. ^22^ But in the Serbian version students who had older family members displayed much more “ageist” attitudes than those who did not, for one particular question. ^23^

Statistical significance was seen in component 1 (Barriers/concerns in dental treatment of elderly) where residents showed more agism than final year undergraduate students. Similar result is seen in Greek version where ‘Cost’ is the Barrier. ^19^ For components 1 (“negative opinion of older people”) and 3 (“oral health care education”), the students from the three years of study had significantly different results in the French version. ^22^ Two items (Q18 and Q27) in factor one (“Negative view of older adults’ life and dental treatment”) showed differences based on the study semester in the Serbian version. ^23^ These differences among the various versions of the agism scale can be attributed to different cultural backgrounds.

When comparing urbanity differences, urban group showed more agism than the non-urban group for the component of ‘time consuming’. Statistical significance was seen in rural and non-rural regarding ‘Value/ethics about older people’ and ‘Elderly patient being more appreciative of care’ in the Greek version of the scale. ^19^ No such difference was seen in the Romanian version ^9^ and Serbian version. ^23^

### Component 1: barriers/concerns in dental treatment of elderly

There were 4 items from the agism scale under component 1 which are 16, 17, 8 and 19 in descending order of factor loading. Item 16 was also seen in the dual institution validation study in USA version of agism scale under the component “Preconceived notions about dental treatment”, ^10^ under “Values/ethics about older people” in the Greek version ^19^ and under the same category as this study in the German version, ^20^ in component 2 (“education around older patients care”) of the French version ^22^ and factor 1 (“Negative view of older adults’ life and dental treatment”) of the Serbian version. ^23^ The Q17 was “whether elderly patients make it long enough to make investment worthwhile” and was seen in the US version under “Preconceived notions about dental treatment”, ^10^ under the component “negative view of older patients” in Brazil version, ^21^ under “Values/ethics about older people” in the Greek version, ^19^ component 2 (“education around older patients care”) of the French version ^22^ and factor 1 (“Negative view of older adults’ life and dental treatment”) of the Serbian version. ^23^

‘If the elderly are better off in old age homes?’ was the eighth question in the agism scale and it was seen under the components “negative view of older patients” in Brazil version, ^21^ under “Values/ethics about older people” in the Greek version ^19^ and “Patient compliance” factor in German version. ^20^ The last item in this component in this study was Q19 which was seen in complexity of providing care for older adults” in Brazilian version, ^21^ under “Barriers to dental care” factor in Greek version, ^19^ “Practitioner’s perspective” in German version ^20^ and factor 1 (“Negative view of older adults’ life and dental treatment”) of the Serbian version. ^23^

### Component 2: patient compliance

This component had 3 items; 5, 6 and 9 where all three were reverse-coded so that higher score indicated more agism. The Q5 which asked if they pay more attention to their elderly patients was also seen in the Brazilian version of agism scale under the component “Positive view of older adults”, ^21^ under “Dentist–older patient interaction” in the Greek version, ^19^ component “Perceptions of the older and their value in society” in the Romanian version ^9^ and “Dental care in younger and older patients” in the Serbian version. ^23^ The question 6 was seen in component “Positive view of older adults” in Brazilian version, ^21^ under “Dentist–older patient interaction” in the Greek version, ^19^ under “Opinions about elderly” in the German version, ^17^ “Perceptions of the older and their value in society” in the Romanian version ^9^ and “Dental care in younger and older patients” in the Serbian version. ^23^ The item 9 which was regarding elderly patients being more appreciative of the care provided was seen in the component Dentist–older patient interaction” in the Greek version ^19^ and “Patient compliance” factor in German version. ^21^

### Component 3: opinions of elderly

The questions 13 and 14, were under this component. They were also seen in the component “Patient compliance” in USA version ^10^ and Greek version, ^19^ under “negative view of older patients” in the Brazil version, ^21^ “general negative view” in the French version ^22^ and “ethical values about older people and patient compliance” in the Serbian version. ^23^

### Component 4: time consuming

The Q2 which was regarding the opinion that taking medical history from elderly patients was time consuming and Q3 being that taking medical history was complex were present in this component of our study, in the Brazilian version of agism scale under “complexity of providing care for older adults” ^21^ and “difficulties in medical history taking” in the Serbian version. ^23^

### Component 5: treatment success rate

The items 12 and 21 were grouped in the ‘Treatment success rate’ component. In the German version Q12 was seen under the component “Opinions about elderly” and Q21 was seen in “Barriers/concerns on dental treatment in elderly” ^20^ and under “Exposure to geriatric dental training and experiences” in the Romanian version. ^9^

### Component 6: practitioner perspective

The questions 24 and 25 in this component were reversed so that higher score indicated more agism. Q24 was not seen in any of the versions of the agism scale and Q25 was seen Under “Barriers to dental care” in the Greek version, ^19^ in “education around older patients care” of the French version ^22^ and under “barriers to dental treatment” in the Serbian version. ^23^

As seen above, questions 13, 14, 16 and 17 are included in the dual institution validation of agism scale carried out in USA ^10^ but under different components than seen in our study. In the Greek version questions 5, 6, 8, 13, 14, 16, 17, 19 and 25 were included in the scale where Q19 is under the same factor “Barriers/concerns”. ^19^ The questions that were included in the German version were 6, 8, 9, 12, 16, 19 and 21 among which Q9 and Q16 were under same components of “Patient Compliance” and “Barriers/Concerns”. ^20^ In the Brazilian versions the similar questions included were 2, 3, 5, 6, 8, 13, 14, 17 and 19 but none of the components were similar. ^21^ Only three questions *i.e.*, 5, 6 and 21 were similar in the Romanian version of agism scale. ^9^ The following five questions were present in the French version: Q16, 17, 13, 14, and 25. ^22^ Highest similarity was seen with the Serbian version as the questions 2,3,5,6,8,13,14,16,17 and 25 were included in their version of agism scale and Q8, 16 and 17 were under the component “Negative view of older adults’ life and dental treatment”. ^23^ In this study it was included in “barriers/concerns in dental treatment of elderly” indicating that though named differently they point towards the negative aspect of treating an elderly patient. Although the results of this study are comparable to those of other studies on the ageism scale for a diverse nation like ours, generalizability is constrained.

### Limitations

Since this study is done on a single institution the generalizability of its result is limited. In-spite of being statistically significant the differences are small and interpretation must be done cautiously. Similar studies of validation should be carried out in different geographical and cultural areas which can help in constructing a single scale for our country.

## Conclusions

Preliminary validation resulted in a 15-item scale with 6 components with acceptable validity of the agism scale and could be further tested in large samples. The female participants had more concern for the elderly patients but had an opinion that treating them would be time consuming. The residents expressed more barriers/concerns while treating the elderly and urban students felt it was more time consuming.

These results necessitate the need for educating the dental students regarding care towards the elderly patients. This scale will help recognise agism in Indian context and provide necessary information to make changes in the curriculum as required.

## Data Availability

Figshare. Responses of dental students to ageism scale: India. https://doi.org/10.6084/m9.figshare.21931383.v2. ^15^ This project contains the following underlying data: Ageism scale for dental student. (This dataset contains consent of the participants. It contains demographic data such as gender, age, year of study, months of clinical experience, attending gerodontology course, presence of elderly member in the family and area of residence. It also depicts the responses of dental students to each of the 27 items in the ageism scale.). This project contains the following extended data:
- Ageism Scale consent form (This document contains information sheet both to the subject experts and the participants. It also contains the consent form.) https://doi.org/10.6084/m9.figshare.22001531.v1. ^14^
- Agism Scale Questionnaire (This document contains survey instrument which is the ageism scale for dental students for both the subject experts and students. It also contains questions on demographic data.) https://doi.org/10.6084/m9.figshare.22001510.v1. ^13^
- STROBE Checklist (This file contains data of STROBE checklist for this article. The corresponding page numbers for the questions in the checklist are mentioned accordingly.) https://doi.org/10.6084/m9.figshare.22121153.v1 Data are available under the terms of the Creative Commons CC0 1.0 Universal (CC0 1.0) Public Domain Dedication.
