# Supplementary material for: Validation of ageism scale for dental students in India: (Ageism Scale for Dental Students- India) – a cross sectional study
Source: F1000Res. 2023 Jul 19;12:413. Originally published 2023 Apr 18. [Version 2] doi: 10.12688/f1000research.130685.2 (PMC10407454; doi:10.12688/f1000research.130685.2)
Supplement: Peer review history

# Peer review: Reviewer response for version 2

**DOI:** 10.5256/f1000research.153142.r188554

**Published:** 2023-08-07

**Authors:** Parvati Iyer

**Affiliations:** 1 Diagnostic Sciences, Arthur Dugoni School of Dentistry, University of the Pacific, Stockton, California, USA

Dear authors,

 The revised article provides clarity and has a better flow of content, improving readability.

 Thank you,

 Reviewer

Is the work clearly and accurately presented and does it cite the current literature?

Yes

If applicable, is the statistical analysis and its interpretation appropriate?

Yes

Are all the source data underlying the results available to ensure full reproducibility?

Yes

Is the study design appropriate and is the work technically sound?

Yes

Are the conclusions drawn adequately supported by the results?

Yes

Are sufficient details of methods and analysis provided to allow replication by others?

Yes

Reviewer Expertise:

Educational research, curricular innovation, cultural competency, critical thinking skills

I confirm that I have read this submission and believe that I have an appropriate level of expertise to confirm that it is of an acceptable scientific standard.

# Peer review: Reviewer response for version 1

**DOI:** 10.5256/f1000research.143457.r179877

**Published:** 2023-07-13

**Authors:** Parvati Iyer

**Affiliations:** 1 Diagnostic Sciences, Arthur Dugoni School of Dentistry, University of the Pacific, Stockton, California, USA

This is such an interesting topic and is very relevant for young dental graduates! Here are my thoughts:

 The paper concludes that the preliminary validation of the ageism scale for dental students in India was successful. The scale can be further tested in larger samples to enhance its validity and reliability. The findings highlight the importance of addressing ageism in dental education and making necessary changes to the curriculum. By recognizing ageism, dental schools can promote a more inclusive and compassionate approach to geriatric dentistry.

**Insights and Suggestions for Improvement:**

 The paper provides valuable insights into the prevalence of ageism among dental students in India and the need for a validated ageism scale. The study contributes to understanding ageism and its implications for dental education and patient care. Here are some suggestions for improvement:

1. Clarify the research objectives: The paper could provide a clearer statement of the research objectives at the beginning to guide readers and enhance the overall coherence of the study.
2. Provide more details on the participants: It would be helpful to provide more information about the participants, such as their age range, demographics, and any potential biases in the sample selection process.
3. Expand the discussion on cultural influences: The paper briefly mentions cultural influences on ageism but could further explore how cultural factors specific to India might shape dental students' perceptions of ageism. This could enhance the understanding of ageism within a cultural context.
4. Consider implications for dental education: While the paper briefly mentions the need for changes in the curriculum, it could expand on the specific areas where dental education can address ageism, such as communication skills, ethical considerations, and patient-centered care for older adults.
5. Provide recommendations for future research: The paper could offer suggestions for future research, such as exploring the relationship between ageism and the quality of dental care provided to older adults or investigating interventions to reduce ageism among dental students.

By addressing these points, the paper can enhance its clarity, depth, and practical implications, thereby contributing further to the field of geriatric dentistry and combating ageism in dental education.

Is the work clearly and accurately presented and does it cite the current literature?

Yes

If applicable, is the statistical analysis and its interpretation appropriate?

Yes

Are all the source data underlying the results available to ensure full reproducibility?

Yes

Is the study design appropriate and is the work technically sound?

Yes

Are the conclusions drawn adequately supported by the results?

Yes

Are sufficient details of methods and analysis provided to allow replication by others?

Yes

Reviewer Expertise:

Educational research, curricular innovation, cultural competency, critical thinking skills

I confirm that I have read this submission and believe that I have an appropriate level of expertise to confirm that it is of an acceptable scientific standard, however I have significant reservations, as outlined above.

# Response

**Published:** 2023-07-17

**Authors:** PRAVEEN JODALLI

**Affiliations:** Manipal University, India

Dear reviewer, thank you for the suggestions

 1. “Clarify the research objectives: The paper could provide a clearer statement of the research objectives at the beginning to guide readers and enhance the overall coherence of the study.”

 The research objectives were to quantify the content validity of the ageism scale by subject experts and measure the construct validity, structural validity, and internal consistency of the ageism scale for dental students by final year undergraduate dental students and residents studying in one of the dental colleges of India.

 2. “Age range, demographics, potential bias in sample selection”

 These parameters already explained in Table 1 and under study population

 3. “Expand the discussion on cultural influences: The paper briefly mentions cultural influences on ageism but could further explore how cultural factors specific to India might shape dental students' perceptions of ageism. This could enhance the understanding of ageism within a cultural context.”

 Assessing the cultural influence on ageism was not the objective of the study and the same is also mentioned in the limitations.

 4.“Provide recommendations for future research: The paper could offer suggestions for future research, such as exploring the relationship between ageism and the quality of dental care provided to older adults or investigating interventions to reduce ageism among dental students”

 The following is added “The paper could offer suggestions for future research, such as exploring the relationship between ageism and the quality of dental care provided to older adults to reduce ageism among dental students” along with the existing recommendation” Similar studies of validation should be carried out in different geographical and cultural areas which can help in constructing a single scale for our country.”

 5. “Consider implications for dental education: While the paper briefly mentions the need for changes in the curriculum, it could expand on the specific areas where dental education can address ageism, such as communication skills, ethical considerations, and patient-centered care for older adults”

 This paper provides reasons for addition of geriatric dentistry in dental curriculum and to have a single window to treat older adults. This is to ensure that future oral health practitioners will have adequate knowledge, skill and empathy while treating older adults.

# Peer review: Reviewer response for version 1

**DOI:** 10.5256/f1000research.143457.r170036

**Published:** 2023-06-23

**Authors:** Chandrashekar Byalakere Rudraiah (B. R)

**Affiliations:** 1 Department of Public Health, JSS Dental College and Hospital, JSS Medical Institutions Campus, Jagadguru Sri Shivarathreeshwara University, Mysuru, Karnataka, India

This article focussing on validating an ageism scale for dental students in Indian context was very essential keeping in mind the lack of such scale for assessing the perception of dental students who will face the task of treating geriatric patients in their routine practice. The scale to assess perception of dental students in India becomes more important as we do not have a specialized subject of gerodontology in the existing curriculum prescribed by dental council of India.

**Strengths of the article: **The introduction highlights the gap in Indian context while citing relevant literature on importance of having such customized scale for dental students. The methodology used is described in detail so that the study is replicable. The factor analysis (Principal component analysis) and other statistical methods for assessing the reliability and validity of the tool are explained and are relevant and appreciate. The results are rationally interpreted and discussed by citing existing relevant literature.

**Novelty:** This is the first of its kind scale that is customized for assessing perceptions of graduating dental students in Indian context. The socio-cultural differences between different regions of the world makes it essential to validate any scale to the local context, which was attempted in this research.The scale helps in eliciting differences in perceptions in relation to socio-demographic characteristics.

**Recommendation for future studies: **A mixed method research design using this questionnaire in quantitative research along with in-depth interviews/focus group discussions will increase validity evidence. Response process validity could be elicited using retrospective verbal probing technique on a sample of patients. Known group validity evidence can be assessed by comparing the scores in existing study with a group of senior dental professional who are trained in gerodontology.

**Expected outcome: **The article highlights the importance of a customized scale for Indian dental graduates and perhaps may eventually lead to the mandatory establishment geriatric oral health units in all dental colleges in the country. The training on geriatric oral health by a multidisciplinary team of experts under one roof will be an eventual anticipated outcome if future studies are conducted using this scale which is validated to Indian context.

 Overall, it is a good tool to be used further in different settings to get additional validity evidence.

Is the work clearly and accurately presented and does it cite the current literature?

Yes

If applicable, is the statistical analysis and its interpretation appropriate?

Yes

Are all the source data underlying the results available to ensure full reproducibility?

Yes

Is the study design appropriate and is the work technically sound?

Yes

Are the conclusions drawn adequately supported by the results?

Yes

Are sufficient details of methods and analysis provided to allow replication by others?

Yes

Reviewer Expertise:

I am a specialist in Public Health Dentistry and I have worked in assessing the oral health status of populations in rural, urban, and tribal areas including the geriatric population.

I confirm that I have read this submission and believe that I have an appropriate level of expertise to confirm that it is of an acceptable scientific standard.
